# Fate of selenium in biofortification of wheat on calcareous soil: an isotopic study

**DOI:** 10.1007/s10653-021-00841-1

**Published:** 2021-02-25

**Authors:** Saeed Ahmad, Elizabeth H. Bailey, Muhammad Arshad, Sher Ahmed, Michael J. Watts, Scott D. Young

**Affiliations:** 1 Division of Agricultural and Environmental Sciences, School of Biosciences, University of Nottingham, Sutton Bonington Campus, Loughborough, Leicestershire LE12 5RD UK; 2 Pakistan Agricultural Research Council – Mountain Agricultural Research Centre, Gilgit-Baltistan, Pakistan; 3 British Geological Survey, Centre for Environmental Geochemistry, Inorganic Geochemistry, Nottingham, NG12 5GG UK

**Keywords:** Selenium, Biofortification, Wheat, Stable isotopes, Residual Se

## Abstract

Selenium (Se) biofortification of staple cereal crops can improve the Se nutritional status of populations. A field trial employing an enriched stable isotope of Se (^77^Se) was undertaken over three consecutive cropping seasons in a coarse-textured, calcareous soil in Gilgit-Baltistan, Pakistan. The objectives were to (1) assess the feasibility and efficiency of Se biofortification, (2) determine the fate of residual Se, and (3) assess the consequences for dietary Se intake. Isotopically enriched ^77^Se (^77^Se_Fert_) was applied, either as selenate or as selenite, at three levels (0, 10, and 20 g ha^−1^) to a wheat crop. Residual ^77^Se_Fert_ availability was assessed in subsequent crops of maize and wheat without further ^77^Se_Fert_ addition. Loss of ^77^Se_Fert_ was c.35% by the first (wheat) harvest, for both selenium species, attributable to the practice of flood irrigation and low adsorption capacity of the soil. No ^77^Se_Fert_ was detectable in subsequent maize or wheat crops. The remaining ^77^Se_Fert_ in soil was almost entirely organically bound and diminished with time following a reversible (pseudo-)first-order trend. Thus, repeat applications of Se would be required to adequately biofortify grain each year. In contrast to native soil Se, there was no transfer of ^77^Se_Fert_ to a recalcitrant form. Grain from control plots would provide only 0.5 µg person^−1^ day^−1^ of Se. By contrast, a single application of 20 g ha^−1^ Se^VI^ could provide c. 47 µg person^−1^ day^−1^ Se in wheat, sufficient to avoid deficiency when combined with dietary Se intake from other sources (c. 25 µg day^−1^).

**Supplementary Information:**

The online version contains supplementary material available at 10.1007/s10653-021-00841-1.

## Background

Selenium (Se) is a crucial dietary micronutrient for human health, but half a billion people worldwide are at risk of Se deficiency (Combs 2001; Fairweather-Tait et al. 2011; Ligowe et al. 2020a). It is a vital component of many selenoproteins (Brown and Arthur 2001; Antonyak et al. 2018) which play an important role in regulating various body functions, such as metabolism of thyroid hormones and protecting cells from damage by free radicals (Rayman 2000, 2012; Yang et al. 2017). Its deficiency is associated with various health disorders such as cardiovascular diseases, cancer, and reduced fertility (Tinggi 2008; Fairweather-Tait et al. 2011; Zhang et al. 2020). The main sources of Se for humans and animals are foods such as cereals, poultry, meat, and fish; contributions from drinking water and other non-food sources are nominal (Rayman 2008; Joy et al. 2015a, b).

In intermediate and low-income countries, cereals provide a large proportion (50–80%) of daily calorific intake (WHO 2019). In Pakistan, cereals, primarily wheat, account for 75% of the energy supply in an average daily diet (Zia et al. 2014). However, it is suspected that the Se concentration in wheat from Gilgit-Baltistan is normally insufficient to meet the WHO-recommended daily allowance (RDA) of Se (50–70 µg day^−1^) for an adult (Ahmad 2020). The average concentration of Se in locally grown wheat has been reported to be 29 µg kg^−1^ (Ahmad 2020) which would supply < 20% (8.67 µg Se day^−1^) of the Se RDA. Deficiency of Se in human populations can be addressed in multiple ways, such as taking Se supplements, dietary diversification, food fortification, and crop biofortification through agronomic or genetic interventions (White and Broadley 2009; Broadley et al. 2006, 2010; Chilimba et al. 2011). Most of these strategies have various shortcomings associated with them, while the efficacy of others, such as crop improvement and genetic modification, is not yet clear (White and Broadley 2009; White 2016). However, in the case of Pakistan, particularly Gilgit-Baltistan where the population is largely dependent on indigenous agricultural produce (Rasul and Hussain 2015), crop biofortification is the most feasible approach. Furthermore, crop biofortification with fertilisers can easily enhance plant Se content (Broadley et al. 2010; Mao et al. 2014) and has been tested successfully in other countries, such as the UK, Finland, and Malawi (Broadley et al. 2010; Alfthan et al. 2015; Ligowe et al. 2019). Finland adopted crop biofortification in the 1980s with Se-enriched fertilisers and successfully enhanced Se concentration in the Finnish food supply (Broadley et al. 2006; Alfthan et al. 2015). The efficiency of Se biofortification is likely to vary with climatic conditions, agricultural practices, and soil type (Ebrahimi et al. 2019). Therefore, experience gained in other countries may not be applicable to the study area of this project.

Inorganic Se species, selenite (Se^IV^) and selenate (Se^VI^), are both available for plant uptake (Broadley et al. 2006; Li et al. 2008). Selenate is normally used for biofortification because it is more soluble and hence more bioavailable (Chilimba et al. 2012a; Ligowe et al. 2020b), but it is also more prone to leaching, particularly in coarse-textured soils at high pH. It is recognised that soils in Gilgit-Baltistan are largely coarse-textured and calcareous with a high pH (> 7) (Hashmi and Shafiullah 2003). Selenite is normally less efficient in biofortification as it is sorbed strongly by soil Fe oxides and rapidly transferred to humus-bound forms (Li et al. 2008; Ligowe et al. 2019). However, adsorption on Fe oxides in calcareous soils is likely to be weak considering the likely trend in the Se^IV^ adsorption envelope (H_2_SeO_3_, HSeO_3_^−^, SeO_3_^2−^; pKa_1_ = 2.47, pKa_2_ = 7.31). It is possible that high pH might ensure continued bioavailability of ‘residual’ Se—i.e. fertiliser-derived Se (Se_Fert_) remaining in the soil for following crops in subsequent growing seasons.

This study aimed to understand the efficiency of Se biofortification in cereal crops with a single application of Se inorganic species (Se^IV^ and Se^VI^). The objectives of this study were to: (1) study the feasibility and efficiency of Se biofortification using a single application to wheat of an enriched ^77^Se stable isotope to discriminate between soil-derived and fertiliser-derived Se (^77^Se_Fert_); (2) evaluate the fate of residual ^77^Se_Fert_ in a cereal rotation (wheat–maize–wheat), as practised in Gilgit-Baltistan; and (3) assess the consequences of biofortification for dietary Se intake.

## Methods

### Overview

A rotational field trial was undertaken over three consecutive cropping seasons (2017–2018, 2018–2019) in Gilgit-Baltistan. The crops chosen were wheat followed by maize and then wheat in the third growing season. The inorganic Se species, selenite (Se^IV^) and selenate (Se^VI^), were applied as isotopically enriched ^77^Se; each species was applied at three different levels (0, 10, and 20 g ha^−1^) to the first wheat crop in March 2018. After wheat harvest, a maize crop was planted on the site (June 2018), grown, and subsequently sampled (November 2018); a second wheat crop was then planted (December 2018) and harvested (June 2019) as before. Soil was sampled at each harvest and analysed for soluble (Se_sol_), adsorbed (Se_ads_), organic (Se_TMAH_), and total ^77^Se (^77^Se_T_); plant analysis included grain and other parts (Mathers et al. 2017; Ligowe et al. 2020b).

### Site selection and management

An agricultural field at the Mountain Agriculture Research Centre (MARC) Gilgit station (35.68157 N, 74.62981 E) was selected (supplementary material Fig. A1). The total area of the experimental field was 268 m^2^ (17.6 × 15.2 m). Twenty plots (2 m × 2 m) were established with 0.4 m between consecutive plots in the central 178 m^2^ (14.6 × 12.2 m) of the experimental field. A discard area of 3 m was established on all four sides of the selected area which was cultivated in the same way as the rest of the field. Four replicates of each treatment were randomly distributed in a randomised block design. All plots received the same irrigation water and basal fertiliser of 140 kg ha^−1^ nitrogen and 80 kg ha^−1^ phosphorus as per local practice. Before sowing the first wheat crop, the soil was ploughed and seeds were sown by hand in straight lines in each plot according to the local agriculture practice. However, for sowing the second (maize) and third (wheat) crop the soil was not ploughed and seeds were planted with minimum tillage in all the selected areas.

### Preparation and application of ^77^Se^IV^ and ^77^Se^VI^ solutions

Enriched ^77^Se treatment solutions of both ^77^Se^IV^ and ^77^Se^VI^ were prepared from an isotopically enriched stock of elemental ^77^Se (150 mg; 99.66% atom % of ^77^Se), purchased from Isoflex, San Francisco, USA, according to the methods described in Mathers et al. (2017). The treatment solution for each field was applied at the early stem extension stage (Zadoks stage 31). The experimental field was flood-irrigated, and two days later when the field was still moist, Se treatment solutions were applied to each plot. For ^77^Se application, each treatment solution was separately mixed with 6 L irrigation water in a watering can and then sprayed evenly over the plot (1.5 L m^−2^). Each treatment application was followed by spraying with 6 L (1.5 L m^−2^) irrigation water from the same container to wash the treatment solution off the crop leaves.

### Soil and plant sampling and processing

Soil sampling was undertaken before the experiment started (H0, November 2017) and at the end of each growing season, i.e. following the first wheat harvest (H1, June 2018), the maize harvest (H2, November 2018), and the second wheat harvest (H3, June 2019). A five-point composite sample of topsoil (0–20 cm) was collected from each plot using a stainless steel auger. The soil was air-dried and sieved (< 2 mm), and 10 g of each soil sample was finely ground in an agate ball mill (Retsch PM 400, Haan, Germany) for elemental analysis.

At the end of each growing seasons (H1, H2, and H3), plants were harvested from the central 1 m^2^ of each plot (2 m × 2 m) at 5 cm above the ground with a scythe. Plants were subsampled (10% of the total) and then air-dried at the MARC Laboratory in Gilgit. Wheat ears were hand-threshed to separate grain and chaff. Maize plants were divided into stems, leaves, husks, and grain. All of the crop samples were separately milled using an ultra-centrifugal mill fitted with a 0.5-mm stainless steel sieve.

### Soil characterisation

Soil pH was measured using a pH meter (Hanna, model pH 209) with combined glass electrode on a soil–water suspension with a ratio (w/v) of 1:2.5 after shaking end-over-end for 30 min (Rowell 1994). Oxides of Fe, Mn, and Al in soil samples were determined in citrate–bicarbonate–dithionate (CBD) extracts of finely ground soil using a single quadrupole ICP-MS (model iCAP-Q, Thermo Scientific, Bremen, Germany). The milled soil was also used for measuring soil organic carbon in a Leco TruMac CN analyser (Stockport, UK). Acid digestion (HNO_3_–HClO_4_–HF) of finely ground soil was undertaken in PFA vessels using a teflon-coated graphite block digester (Model A3, Analysco Ltd.) controlled by a Eurotherm unit (Mather et al. 2017; Ligowe et al. 2020b). Total selenium concentration (Se_T_), ^77^Se isotopes, and other elemental analyses were undertaken using a triple-quadrupole ICP-MS (model iCAP-TQ, Thermo Scientific, Bremen, Germany).

A three-stage sequential extraction of < 2 mm sieved soil was undertaken with (1) potassium nitrate (0.01 M KNO_3_) followed by (2) potassium dihydrogen phosphate (0.016 M KH_2_PO_4_), and finally (3) 10% tetramethylammonium hydroxide (TMAH) to determine, respectively, ‘soluble’ (Se_sol_), ‘adsorbed’ (Se_ads_), and ‘organic’ (Se_TMAH_) fractions of soil-derived Se (Se_Nat_) and residual ^77^Se_Fert_ (Ligowe et al. 2020b). TMAH extracts organically bound Se by mobilising the soluble (fulvic) and colloidal (humic) soil organic fractions and also potentially through alkaline hydrolysis of organic Se. Selenium speciation analysis was undertaken on the soluble and adsorbed fractions using an HPLC unit (Dionex ICS-5000) coupled to the ICP-MS. The chromatography eluent consisted of 4.00 g L^−1^ NH_4_NO_3_, 20 ml L^−1^ methanol, 0.00325 g L^−1^ NH_4_-EDTA, and 12.1 g L^−1^ Tris buffer. The stationary phase used was a Hamilton PRX-100 anion exchange column (100 × 4.1 mm; 5 µm particle size); the eluent flow rate was 1.4 mL min^−1^.

### Plant analysis

Finely ground plant samples (c. 0.2 g) were microwave-digested in 6 ml HNO_3_ (68% *Primar Plus*™ grade). For grain samples, 0.3 g was digested in 3 ml HNO_3_ (70%), 3 ml Milli-Q water, and 2 ml H_2_O_2_ (30%). The final volume of digested sample was made to 20 ml (plant) and 15 ml (grain). Each digestion batch included nine operational blanks and a certified reference material (CRM) (rice flour standard, NIST 1568b). The mean recovery of Se in the CRM (NIST 1568b) was 96% (certified value: 365 µg kg^−1^, measured value: 351 ± 1.67 µg kg^−1^, *n* = 9). A 1:10 dilution with Milli-Q water was done prior to analysis of Se isotopes and multi-elemental analysis via ICP-MS (iCAP-TQ).

### Processing TQ-ICP-MS ^77^Se and ^80^Se intensity data

The raw intensity data of Se isotopes (^77^Se and ^80^Se) were exported as intensity values (counts-per-second; CPS) from the triple quadrupole ICP-MS (iCAP TQ). Both isotopes were measured in O_2_ cell mode as mass-shifted to m/z 93 (^77^Se^16^O) and m/z 96 (^80^Se^16^O) to reduce interferences from Se and Ge hydrides and the ^40^Ar dimer. The ^77^Se intensity signals were also corrected for minor interference at mass 93 (^76^Ge^1^H^16^O) by running a Ge standard (5 µg L^−1^). Drift correction relied on Rh as an internal standard. Calibration slopes for both isotopes (^77^Se and ^80^Se) were derived from multi-isotope Se calibration standards (SPEX CertiPrep CLMS-2; 1, 2, 5, and 10 µg L^−1^). The concentration of native ^77^Se in each sample was calculated from the natural isotopic abundance of ^77^Se and the measured concentration of total (native) Se (from ^80^Se); the ^77^Se derived from fertiliser (Se_Fert_) was then obtained by difference.

### Modelling the loss of Se_Fert_

The loss in Se_Fert_ concentration as a function of time was described using a reversible first-order equation (Eq. 1.), adapted from Crout et al. (2006), in which Se_t_ is Se_Fert_ remaining in soil at time *t*, Se_0_ is the original concentration of Se_Fert_ added to the soil (g ha^−1^), Se_Eq_ is the ‘equilibrium’ Se_Fert_ remaining at infinite time, and K is the sum of the forward and reverse rate constants (*k*_1_ + *k*_2_).

1 $${{Se}}_{{{t}}} = {{ Se}}_{{{{Eq}}}} + \left( {{{Se}}_{0} - {{Se}}_{{{{Eq}}}} } \right) \exp \left( { - Kt} \right).$$

### Calculation of distribution coefficient

The distribution coefficient (*kd*) is the ratio of Se_ads_ to Se_sol_ and was calculated for both Se_Nat_ and Se_Fert_, respectively, from Eq. 2:

2 $$kd = \frac{{{{Se}}_{{{{ads}}}} }}{{{{Se}}_{{{{sol}}}} }}$$

where Se_ads_ and Se_sol_ are the concentrations of Se in soil (µg kg^−1^) in the ‘adsorbed’ and ‘soluble’ fractions.

### Calculating bioconcentration factor and bioavailability ratio

The bioconcentration factor (BCF) is formulated as the ratio of Se_Nat_ or Se_Fert_ in the plant to their respective concentrations in soil and is a convenient index of bioavailability. The BCF values for the wheat crop at H1 for both species of Se_Fert_ (Se^IV^ and Se^VI^) and Se_Nat_ were calculated from Eq. 3:

3 $${{Se}}_{{{{BCF}}}} = \frac{{{{Se}}_{{{{plant}}}} }}{{{{Se}}_{{{{soil}}}} }}.$$

In Eq. 3, Se_plant_ is Se concentration (µg kg^−1^) in each fraction (straw, chaff and grain) of wheat plant, respectively, and Se_soil_ is total Se_Nat_ or Se_Fert_ concentration (µg kg^−1^) in the corresponding soils. The total soil Se_Nat_ concentration used in Eq. 3 was measured in the soil HF digests, while the total Se_Fert_ concentrations in soil of 4 and 8 µg kg^−1^ were calculated from the application rates of 10 and 20 g ha^−1^, respectively, assuming a topsoil mass of 2500 t ha^−1^.

The *relative* bioavailability of Se_Fert_ (Se^IV^ and Se^VI^) and Se_Nat_ in the H1 wheat crop was calculated as a ‘bioavailability ratio’ (*B*_R_) from Eq. 4:

4 $$B_{{{R}}} = \frac{{{{BCF}}_{{{{Fert}}}} }}{{{{BCF}}_{{{{Nat}}}} }}$$

where BCF_Fert_ represents the bioconcentration factor for fertiliser-derived Se (^77^Se_Fert_) and BCF_Nat_ is the bioconcentration factor for soil-derived Se (Se_Nat_) in the plant.

### Statistical analysis

Basic statistical calculations including mean, median, standard deviation, and standard error were performed in Microsoft Excel 2016, while Minitab (version 18.1) was used for the ANOVA.

## Results and discussion

### Soil characteristics

Basic soil characteristics including soil pH and the concentrations of organic carbon, CaCO_3,_ and metal oxides (Fe_2_O_3_, MnO_2,_ and Al(OH)_3_) were similar across all the field plots and did not vary significantly (ANOVA, *p* > 0.05). The mean soil pH of all plots was 7.84 ± 0.05. The soil organic carbon and CaCO_3_ contents were 1.60% ± 0.103 and 1.73% ± 0.270, respectively; the mean concentrations of Fe, Mn, and Al oxides were 3.13, 0.151, and 0.738 g kg^−1^, respectively. The mean total soil Se and TMAH-extractable Se concentrations were 139 µg kg^−1^ and 94 µg kg^−1^, respectively, suggesting a very low overall Se concentration and the presence of a non-organic recalcitrant Se phase (c. 45 µg kg^−1^).

### Fertiliser Se dynamics in soil

**Fig. 1:**
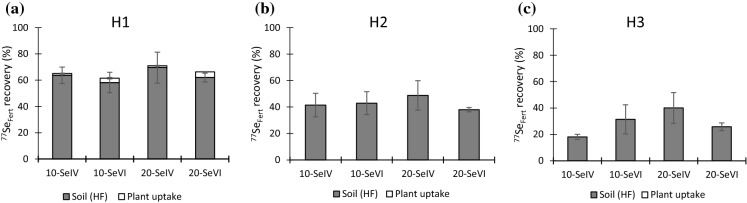
Fate of ^77^Se_Fert_ in soil after **a** first wheat harvest (H1), **b** maize harvest (H2), and **c** second wheat harvest (H3). Error bars represent standard error of means (*n* = 4)

The concentration of fertiliser-derived Se (Se_Fert_) in soil decreased with time following its application in March 2018 (Table 1). Compared to its original application of 10 and 20 g ha^−1^, the average concentration of Se_Fert_ had decreased by 30% and 42% at wheat harvest (H1; June 2018), by 51% and 62% at maize harvest (H2; November 2018) and by 60% and 82% at the second wheat harvest (H3; June 2019) (Fig. 1). There was no significant difference in the proportion of Se_Fert_ lost between the four treatments at any of the three harvests (ANOVA, *p* > 0.05).

**Table 1:** Status of fertiliser Se (^77^Se_Fert_) in the soil after each harvest (wheat harvest = H1, maize harvest = H2, second wheat harvest = H3). Concentrations presented are the average of four replicate plots

Treatment types and level of application (g ha^−1^)^a^	Plant uptake (g ha^−1^)			^77^Se_Fert_ remaining in soil (g ha^−1^) (percentage recovery in brackets)			Total loss (g ha^−1^)		
	H1	H2	H3	H1	H2	H3	H1	H2	H3
10-Se^IV^	0.136	< LOD	< LOD	6.37 (36.3)	4.15 (58.5)	1.81 (81.9)	3.49	5.85	8.19
10-Se^VI^	0.334	< LOD	< LOD	5.82 (41.8)	4.29 (57.1)	3.14 (68.6)	3.85	5.71	6.86
20-Se^IV^	0.287	< LOD	< LOD	13.9 (30.5)	9.75 (51.3)	8.02 (59.9)	5.80	10.2	12.0
20-Se^VI^	0.864	< LOD	< LOD	12.4 (38.0)	7.61 (62.0)	5.17 (75.2)	6.74	12.4	14.8

^a^The numbers (10 and 20 g ha^−1^) before the treatment type represent the level of ^77^Se_Fert_ application

The decrease in Se_Fert_ concentration at H1 can be attributed to the combined effects of plant uptake and loss of Se from the soil through leaching or volatilisation. The losses due to plant uptake at H1 were low and varied between treatments and application levels. For Se^IV^, removal by the crop was 1.36% and 1.46% at 10 g ha^−1^ and 20 g ha^−1^, respectively; equivalent figures for Se^VI^ were greater, at 3.34% and 4.32%, which reflects the greater bioavailability of selenate. The minor contribution to ^77^Se_Fert_ loss by crop uptake at H1 suggests that ^77^Se_Fert_ was lost either through volatilisation or through leaching in irrigation water due to irrigation shortly after application of ^77^Se_Fert_. It has been reported that volatilisation of Se accounts for a small proportion (6.1%) of Se loss from sediments (Karlson and Frankenberger 1990). Hence, it is reasonable to assume that flood irrigation (widely practiced locally) was the main cause of loss of ^77^Se_Fert_, especially as the soil has a high pH which limits the retention of Se on Fe oxides. Surprisingly, the difference in total loss between Se^IV^ and Se^VI^, mainly due to leaching, was quite small which again may reflect the high pH of the soil at which Se^IV^ adsorption is comparatively weak. Further loss of ^77^Se_Fert_, measured at H2 and H3, was not due to plant uptake because ^77^Se_Fert_ in H2 and H3 crops was below the detection limit for ^77^Se (c. 0.25 µg kg^−1^). Continued losses of ^77^Se_Fert_ from the soil through leaching may be particularly likely in these soils because of their high pH, coarse texture, and low organic carbon content (1.6%); these are all characteristics which will reduce the ability of the soil to retain added Se (Gissel-Nielsen and Hamdy 1977; Moreno et al. 2013; Lopes et al. 2017).

Speciation analysis of post-H1 soils demonstrated that the only inorganic form of ^77^Se_Fert_ present in the soluble and adsorbed fractions in most of the samples was Se^IV^ (supplementary material Table B1). It is reported that approximately 20% of the Se^VI^ applied to Finnish soils is taken up by plants, whereas the remaining Se is reduced to Se^IV^ and immobilised in the soil (Keskinen et al. 2009). The findings of this study are also consistent with Stroud et al. (2010) who studied the fate of Se added as Se^VI^ to soils in the UK and reported that Se^VI^ was not detectable in soil samples either before or after fertiliser application. It was initially thought that the calcareous nature of the soil might ensure the survival of some of the ^77^Se_Fert_ as Se^VI^, at least at H1, but this was not the case.

The added ^77^Se_Fert_ did not significantly increase total soil Se because the addition of 10 and 20 g ha^−1^ would only contribute concentrations of 4 and 8 µg kg^−1^, respectively, assuming 2500 t ha^−1^ of topsoil. By contrast, the average concentration of the native soil Se was 139 ± 9.12 µg kg^−1^.

### Fractionation of selenium

#### *Native soil Se (Se*_*Nat*_*)*

The three-step extraction procedure demonstrated that the fractionation of Se_Nat_ was fairly consistent across all four sampling events (H0, H1, H2, and H3) (supplementary material Fig. A2 and Table B2). The soluble and adsorbed fractions of Se_Nat_ accounted for < 2.5% (0.30–3.27 µg kg^−1^) and < 2% (1.12–2.61 µg kg^−1^), respectively, across all samples. The organically bound Se (Se_TMAH_) was typically constant at 60% (75–88 µg kg^−1^) for Se_Nat_ in H0, H1, and H2 samples but was substantially reduced to 43% (60 µg kg^−1^) in H3 samples. The remaining Se_Nat_ of 40–57% (47.4–78.2 µg kg^−1^) may be regarded as a ‘recalcitrant’ fraction of Se, locked up within mineral structures (Keskinen et al. 2009; Mathers et al. 2015). Possible chemical forms of recalcitrant Se are not known. It is likely that a proportion of Se added to the soil from rock weathering, atmospheric deposition, or irrigation water could be converted to a recalcitrant phase within CaCO_3_; alternatively, it could simply represent Se within parent material, again possibly CaCO_3_, which slowly contributes more reactive fractions of Se due to weathering. Therefore, it is not clear whether the recalcitrant Se represents a sink or a source of bioavailable Se.

#### *Fertiliser Se (*^*77*^*Se*_*Fert*_*)*

The soluble and adsorbed fractions of ^77^Se_Fert_ in post-H1 soils accounted for 8.5–8.6% (0.498–0.545 g ha^−1^) and 5.3–6.3% (0.340–0.365 g ha^−1^), respectively, in 10 g ha^−1^ application treatments, whereas for 20 g ha^−1^ applications the soluble and adsorbed fractions accounted for 7.3–7.9% (0.978–1.020 g ha^−1^) and 4.0–6.1% (0.501–0.849 g ha^−1^), respectively (supplementary material Fig. A3 and Table B3). There was no significant difference in the soluble fractions (Se_sol_) of Se^IV^ and Se^VI^ treatments and the same was observed for the adsorbed fraction (Se_ads_) (ANOVA, *p* > 0.05). The average combined concentrations of soluble and adsorbed fractions for 10 and 20 g ha^−1^ treatments varied with time and decreased to 4.54% (0.297 ± 0.15 g ha^−1^) and 3.03% (0.201 ± 0.11 g ha^−1^) in H2 soils but slightly increased to 9.80% (0.369 ± 0.266 g ha^−1^) and 6.26% (0.254 ± 0.173 g ha^−1^) in H3 soils. As found for H1 soils, there was no significant difference in the native soil soluble or adsorbed fractions of Se^IV^ and Se^VI^ treatments in H2 and H3 soils, respectively (ANOVA, *p* > 0.05).

The results of sequential extraction for ^77^Se_Fert_ and Se_Nat_ demonstrated that a comparatively larger proportion of freshly added Se was present in the bioavailable (soluble and adsorbed) fraction at H1 compared to H2 and H3 soils. However, available ^77^Se_Fert_ was too low to contribute to plant Se uptake in H2 and H3 crops which is consistent with the findings of Gissel-Nielsen et al. (1984) who found a minimal residual availability of Se in pasture systems. Similarly, Chilimba et al. (2012b) and Mathers et al. (2017) reported minimal recovery of residual Se in maize and wheat crops, respectively. Ligowe et al. (2019, 2020b) also reported much lower recoveries of residual Se in maize and green vegetables.

The remaining ^77^Se_Fert_ in H1, H2, and H3 soil samples was all present in an organically bound form; all the remaining ^77^Se_Fert_ was extractable with TMAH, and so there were insignificant concentrations present in a ‘recalcitrant’ pool. The sum of all extractable fractions (soluble, adsorbed, and organically bound) in each sample was equal to the total concentration of Se_Fert_ in the soil after each harvest, but the total concentration decreased with time compared to the original application. An average decrease of 37% was observed in ^77^Se_Fert_ concentration after 72 days (H1) since the initial application; the remaining ^77^Se_Fert_ decreased further to 43% and 29% after 224 days (H2) and 439 days (H3), respectively.

**Fig. 2:**
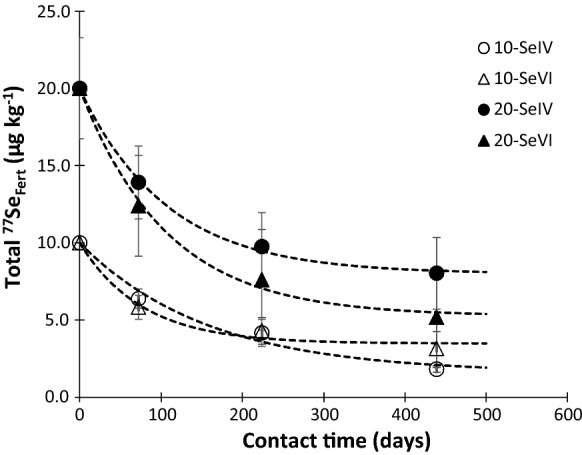
Measured (data points) and modelled (dotted lines) concentration of ^77^Se_Fert_ in soil as a function of its residence time using a reversible first-order kinetics model. The numbers (10 and 20) before Se species (Se^IV^ and Se^VI^) in the legend represent the application rate in g ha^−1^. Error bars represent standard error of means (*n* = 4)

Figure 2 shows the measured soil Se concentrations and modelled data (calculated from Eq. 1). The trend approached a nonzero asymptote within the time frame of the study, suggesting long-term retention of some of the ^77^Se_Fert_. However, as already discussed, the remaining ^77^Se_Fert_ was virtually all present as humus-bound residues (extractable with TMAH) and was not available for plant uptake beyond H1. With limited data (four time points), the trends shown must be interpreted with caution. Application of 20 g ha^−1^ showed the clearest contrast in Se_Eq_ (Eq. 1) between Se^IV^ and Se^VI^ with 40% and 26% remaining in the soil; equivalent values for 10 g ha^−1^ applications were 16% and 35%, but the overall trends were very similar over the 439-day period.

#### Risk assessment of water contamination

The continued loss of ^77^Se_Fert_ from soil to groundwater poses a potential risk of contamination to drinking water in the catchment area. Therefore, a simple risk assessment was undertaken to estimate the loss of Se to groundwater by considering the use of 6 million L ha^−1^ irrigation water applied as 12 irrigation events (50 mm depth). This is a common practice in the area due to the coarse-textured soil (Mountain Agriculture Research Centre, personal communication, August 2019). Approximately 30% (~ 3 and 6 g ha^−1^) of ^77^Se_Fert_ was lost at H1 for the application rates of 10 g ha^−1^ and 20 g ha^−1^ which could therefore result in concentrations of 0.50 µg L^−1^ and 1.0 µg L^−1^ Se in drainage water, respectively, after the wheat-growing season. Alternatively, in a worst-case scenario, if it is assumed that all the ^77^Se_Fert_ applied (10 and 20 g ha^−1^) to the wheat crop was lost with the first irrigation of 0.5 million L ha^−1^, this would result in 20 and 40 µg L^−1^ of Se in drainage water. Both of the above values, 20 and 40 µg L^−1^, are still below the US EPA maximum contamination level of 50 µg L^−1^ for Se in drinking water, and neither of these scenarios allows for the dilution of drainage water that would occur following egress of Se-enriched water from the soil into surface water systems.

#### *Distribution coefficient of Se*_*Nat*_* and *^*77*^*Se*_*Fert*_* in soil*

The *kd* values for Se_Nat_ and ^77^Se_Fert_ were very low with similar mean values of 0.76 ± 0.141 and 0.70 ± 0.199 demonstrating a very low absorption capacity in this soil to retain Se in an adsorbed reactive form. This is expected because the soil has a coarse texture, high pH, and low organic carbon content. The lack of a significant difference in the kd values for Se_Nat_ and ^77^Se_Fert_ (ANOVA, *p* > 0.05) suggests that the added ^77^Se has achieved isotopic equilibrium within the ‘reactive’ Se fractions (Se_sol_ and Se_ads_). When Se is added to soil, some proportion of it will gradually transform into organically bound or recalcitrant phases, but the reactive pools should reach equilibrium rapidly. The low *kd* values and low retention ability of this soil also confirm the necessity for repeated seasonal applications of Se to each crop.

### Selenium in the wheat crop at Harvest 1 (H1)

The application of different levels and species of Se had no effect on crop yield which is consistent with other investigations (Curtin et al. 2008; Broadley et al. 2010; Mathers et al. 2017). The average yields of straw + chaff and grain, based on harvest of the central 1 m^2^ of each plot, were 4.8 and 3.5 t ha^−1^, respectively.

**Fig. 3:**
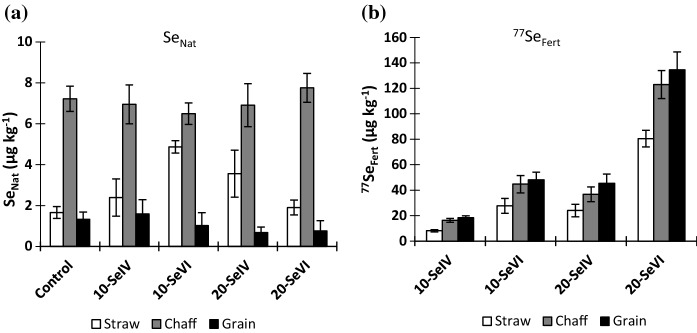
Selenium concentration in plant tissues at Harvest 1 (H1) originating from **a** soil native Se (Se_Nat_) and **b** fertiliser-derived Se (^77^Se_Fert_). Error bars represent standard error of means (*n* = 4). Note the different scales in Fig. 4a, b

The concentrations of Se_Nat_ in the aboveground biomass (sum of straw, chaff, and grain) were very small compared to ^77^Se_Fert_ and similar across all plots (Table 2). The concentration of Se_Nat_ varied between plant tissues (straw, chaff, and grain) in all treatments (Fig. 3). Chaff had the highest average concentration of Se_Nat_ at 7.15 µg kg^−1^ followed by straw (2.87 µg kg^−1^) and grain (1.14 µg kg^−1^) in all cases. The native Se concentration in chaff and grain was constant across all treatments. However, the concentration of Se_Nat_ in straw revealed significant variation between different treatments (ANOVA, *p* < 0.05) (Fig. 3); the reason for this is not clear as there is no reason to expect a difference in Se_Nat_ in different plant tissues caused by the ^77^Se_Fert_ application. Furthermore, similar investigations (Chilimba et al. 2012a, b; Mathers et al. 2017; Ligowe et al. 2020b) in other type of soil have not shown changes in Se_Nat_ concentration in plant tissues across different treatments. However, with Se_Nat_ concentration being so small in all plant tissues it is possible that they may be subject to relatively substantial systematic analytical errors.

**Table 2:** Concentration of Se in wheat (sum of chaff, grain, and straw accounting for their relative masses per unit area) at H1 originating from soil (Se_Nat_) and fertiliser (^77^Se_Fert_), and a plant enrichment factor (the proportional increase in Se in the plant above native levels originating from the application of ^77^Se_Fert_)

Treatments	Plant total Se	Soil derived Se (Se_Nat_)	Fertiliser-derived Se (^77^Se_Fert_)	Plant enrichment factor
	(µg kg^−1^)	(µg kg^−1^)	(µg kg^−1^)	
Control	2.95	2.76	0.188	
10-Se^IV^	17.7	3.25	14.5	6.02
10-Se^VI^	43.9	3.56	40.3	14.9
20-Se^IV^	38.0	3.26	34.7	12.9
20-Se^VI^	116	2.94	113	39.5

The numbers in ‘Treatments’ before Se species (selenite (Se^IV^) and selenate (Se^VI^)) indicate the Se application rate (10 and 20 g ha^−1^)

The concentration of Se was significantly greater in all plant tissues due to fertiliser Se application (Fig. 3). Both species of ^77^Se_Fert_ (Se^IV^_Fert_ and Se^VI^_Fert_) enhanced Se concentration in the wheat plant (grain, chaff, and straw) at harvest, but ^77^Se^VI^_Fert_ was more effective compared to ^77^Se^IV^_Fert_. A single application of 10 and 20 g ha^−1^ of ^77^Se^IV^_Fert_ resulted in a 14- and 32-fold increase in grain Se compared to an extremely low grain Se concentration of 1.42 µg kg^−1^ in control plots. The same application rates of ^77^Se^VI^_Fert_ produced a 35- and 95-fold increase in grain Se over control plots.

The greater efficiency of Se^VI^ in enhancing plant Se content observed in this study is consistent with other investigations, notwithstanding the calcareous nature of the soils in Gilgit-Baltistan. Gupta and Winter (1989) reported that Se^VI^ applications resulted in 5–18 times greater Se concentrations in forages and barley grain compared to the same application of Se^IV^. Chen et al. (2002) reported a ninefold increase in rice Se in China, compared to a 6.6-fold increase, as a result of 20 g ha^−1^ application of Se^VI^ and Se^IV^, respectively. Boldrin et al. (2013) applied Se^IV^ and Se^VI^ to soil, as a foliar spray, and found that Se^VI^ was more effective in raising rice Se concentrations in Brazil for both methods of application. Ros et al. (2016) compared a large set of data on Se biofortification and found that, on average, Se^VI^ was 33 times more effective in increasing plant Se contents compared to Se^IV^ at the same rate of application. However, in the current trial, for each g ha^−1^ of Se^VI^ applied the grain Se concentration was increased by only 4.76–6.70 µg kg^−1^, which is less efficient compared to other investigations. Broadley et al. (2010), Chilimba et al. (2012a), and Mathers et al. (2017), respectively, reported increases in grain Se concentration of 16–26 µg kg^−1^, 15–21 µg kg^−1^, and 12.1–17.3 µg kg^−1^ for each g ha^−1^ of Se^VI^ applied.

#### *Recovery of *^*77*^*Se*_*Fert*_* species in plants at H1*

The recovery of ^77^Se_Fert_ by wheat plants (sum of straw, chaff, and grain) from soil was different for the two Se species (Table 3): selenate recovery was 33–40% greater than that of Se^IV^_Fert_ at application rates of both 10 and 20 g ha^−1^. The average recoveries of Se^IV^_Fert_ by wheat plants were only 1.36% and 1.43% for 10 and 20 g ha^−1^ application, respectively (Table 3); the equivalent recoveries for Se^VI^_Fert_ were 3.34% and 4.32%, respectively. For both species, the recovery was greater at the higher application rate (20 g ha^−1^). The recovery of ^77^Se_Fert_ species also varied between plant tissues (Table 3); grain had a higher recovery in all cases followed by chaff and straw.

**Table 3:** Recovery (% of application) of ^77^Se_Fert_ in different plant tissues at the first wheat harvest (H1). Treatments below indicate the Se application rate (10 and 20 g ha^−1^) and the Se species (selenite (Se^IV^) and selenate (Se^VI^))

Plant tissue	^77^Se_Fert_ recovery in wheat crop (% of application)			
	10-Se^IV^	10-Se^VI^	20-Se^IV^	20-Se^VI^
Straw	0.250	0.797	0.393	1.05
Chaff	0.345	0.821	0.353	1.17
Grain	0.768	1.72	0.686	2.10
Total plant^a^	1.36	3.34	1.43	4.32

^a^‘Total plant’ represents the sum of straw, chaff, and grain; roots were not sampled

Compared to other investigations on field-grown wheat and other cereal crops, the recovery of both species was low. Mathers et al. (2017) reported 25.9–44.5% recovery in wheat plants grown on three contrasting sites in the UK with an application of 10 g ha^−1^ of ^77^Se^VI^. Broadley et al. (2010) observed recoveries of 19.6–34.7% in a wheat crop (grain + straw) following Se^VI^ (aqueous Na_2_SeO_4_) application at six different rates (ranging from 5 to 100 g ha^−1^) in the UK. However, Lyons et al. (2004) reported slightly smaller recoveries of 1.8–9.3% for foliar application of Se^VI^ at four different rates (10, 30, 100, and 300 g ha^−1^) to a wheat crop in Australia. Similarly, Ducsay and Ložek (2006) reported plant uptake of 2.4–9.3% for foliar application of Se to a wheat crop in Slovakia.

The recovery of ^77^Se_Fert_ in wheat grain (first crop) observed for different treatments (Table 3) was smaller than values reported for wheat in the literature: 12.4–15.2% (Mathers et al. 2017), 10–17.3% (Broadley et al. 2010), 12.7–17% (Curtin et al. 2008), 6.5% (Lyons et al. 2004), and 2–6% (Stephen et al. 1989). Recovery was also less than the 6.5–10.8% values reported by Chilimba et al. (2012a) for a maize crop.

The low ^77^Se_Fert_ recoveries in this study compared to other investigations conducted elsewhere could be due to low crop yield, soil characteristics, and possibly a limited period of ^77^Se_Fert_ availability for plant uptake in the Gilgit-Baltistan soils. Soil texture was sandy with low CaCO_3_ and organic carbon contents, which may reduce nutrient retention and thereby render the ^77^Se_Fert_ more prone to leaching after irrigation.

### ***Bioconcentration factor of Se***_***Nat***_*** and ***^***77***^***Se***_***Fert***_

**Fig. 4:**
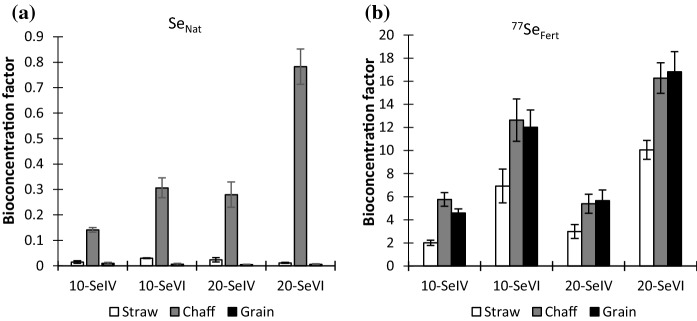
Bioconcentration factor (Eq. 3) for native Se (Se_Nat_) and fertiliser-derived Se (^77^Se_Fert_) in different parts of wheat plants. Error bars represent standard error of means (*n* = 4). Note the different scales in **a**, **b**. The numbers (10 and 20) before the Se species symbol represent the amount of Se applied in g ha^−1^

The values of BCF for ^77^Se_Fert_ (Se^IV^ and Se^VI^) were significantly larger than those of Se_Nat_ (Fig. 4). This is consistent with findings from Mathers et al. (2017) who observed a higher BCF for wheat plants fertilised with Se^VI^ compared to their control plots. Furthermore, as expected, the BCF of ^77^Se^VI^_Fert_ was greater than that of ^77^Se^IV^_Fert_ (Fig. 4) due to greater solubility (Peng et al. 2017). The other reason for differences in BCF between Se^IV^ and Se^VI^ may lie in the mechanisms governing root uptake from soil and subsequent translocation to aerial parts of the plant. Selenate is freely absorbed by plant roots via sulphate transporters and then transported through xylem vessels into plant stems and leaves, whereas a fraction of Se^IV^ is likely to be converted to Se^VI^ or organic Se before being transported to other parts of the plant (White 2016; Ali et al. 2017). The BCF also varied between different parts of the plant in the same treatment as the BCF values for ^77^Se_Fert_ in grain and chaff were similar but substantially greater than for straw due to different abilities of tissue in various parts of the plant to accumulate or retain Se. The other reason for the greater concentration of Se in grain is the transportation of Se in phloem from leaves to grains.

Differences in BCF between ^77^Se_Fert_ and Se_Nat_ and between the applied ^77^Se_Fert_ species of Se^IV^ and Se^VI^ reflect differences in bioavailability. The average B_R_ of Se_Fert_/Se_Nat_ for the H1 wheat crop confirmed the large difference in bioavailability of ^77^Se_Fert_ compared to Se_Nat_ to all tissues of the wheat (Table 4); in particular, the bioavailability of ^77^Se^VI^_Fert_ to wheat grain was 764 and 1261 times that of Se_Nat_ at applications of 10 and 20 g ha^−1^, respectively. The *B*_R_ values of ^77^Se_Fert_/Se_Nat_ were comparable with other Se biofortification investigations (Stroud et al. 2010b; Chilimba et al. 2012a; Galinha et al. 2012) as cited in Mathers et al. (2017) and confirmed the greater bioavailability of ^77^Se_Fert_^VI^ (Table 4). Ali et al. (2017) studied the bioavailability of Se^IV^ and Se^VI^ to wheat plant in a pot experiment and found that mobility and availability of Se^VI^ for plant uptake was 40–90% higher than that of Se^IV^. Similarly, Fan et al. (2015) from his studies on transformation and bioavailability of Se (Se^IV^ and Se^VI^) applied to tobacco plants reported that Se^VI^ was 4.3–7.9 times more bioavailable than Se^IV^.

**Table 4:** Bioavailability ratio (*B*_R_; Eq. 4) in plant tissues of the H1 wheat crop for different concentrations of fertiliser-derived selenite (Se^IV^) and selenate (Se^VI^)

Plant tissue	Bioavailability ratio (*B*_R_) of ^77^Se_Fert_: Se_Nat_			
	10-Se^IV^	10-Se^VI^	20-Se^IV^	20-Se^VI^
Straw	69.4	91.8	72	376
Chaff	34.8	111	41.4	136
Grain	187	764	490	1261

The BR is the ratio of BCF values for added Se (^77^Se_Fert_) to that of soil-derived Se (Se_Nat_)

^*^The number (10 and 20) before the Se species symbol represents the amount of Se applied in g ha^−1^

Values of B_R_ varied between different parts of the plant (Table 4) and were greater in grain than in chaff and straw (ANOVA, *p* < 0.001); there was no significant difference between chaff and straw (ANOVA, *p* > 0.05). Possible reasons for a greater B_R_ in grain lie in the timing, mode of ^77^Se_Fert_ application, and species of ^77^Se_Fert_. The ^77^Se_Fert_ was applied at early stem extension stage, and its greater bioavailability compared to Se_Nat_ would produce a greater concentration of ^77^Se_Fert_ in leaves which is then supplied to grain when its formation begins. The concentration of Se is generally greater in leaves before anthesis and then starts to decrease when its translocation from leaves to reproductive organs begins (White 2016). Furthermore, because ^77^Se_Fert_ was sprayed on the plant canopy it is likely that some of it may have been absorbed into leaves and translocated via phloem from leaves to grain. The B_R_ values for Se^VI^ were greater compared to Se^IV^ because Se^VI^ is readily translocated from roots to other parts of the plant, while Se^IV^ tends to accumulate in roots (Li et al. 2008).

### Consequences of biofortification for dietary Se intake

Wheat is the most important staple crop in Pakistan (Raza 2018) and forms an essential component of the daily diet for the majority (~ 80%) of the local population (Zia et al. 2014). The average daily consumption of wheat-based food in Pakistan is 350 g person^−1^ and accounts for 75% of the daily calorific intake for an individual (Zia et al. 2014). Considering the average daily wheat consumption, the Se concentration in wheat grown on the control plots of this experiment would provide only 0.5 µg person^−1^ day^−1^ which accounts for < 1% of the WHO-recommended (55–70 µg day^−1^) daily Se intake (Table 5). However, the biofortified wheat grain produced in this study with the application of 10 and 20 g ha^−1 77^Se_Fert_^VI^ would provide 17.2 and 47.4 µg day^−1^ accounting for 31.2% and 86.2% of the daily requirement. By contrast, the application of ^77^Se_Fert_^IV^ at the same level would provide only 7 and 16.1 µg person^−1^ day^−1^ contributing 12.7% and 29.3% towards the recommended daily Se intake. Based on Se concentrations in a typical Pakistani food basket from values reported in the literature, the Se contribution from other sources is around 25 µg day^−1^ (Hussain 2001; Iqbal et al. 2008; USDA 2019). Therefore, the consumption of wheat flour obtained from 10 and 20 ha^−1^ Se^VI^ application would increase Se intake to 42.2 and 72.4 µg day^−1^ person^−1^ for individuals with access to diverse food sources, assuming that 25 µg day^−1^ has come from those other sources.

**Table 5:** Grain Se dietary intake resulting from different Se treatments, compared to reliance on native soil-derived Se

Treatments	Se concentration in grain (µg kg^−1^)		Adult daily Se intake from grain (µg person^−1^)		Proportion (%) of recommended adult daily Se intake (RDI)
	Mean	Standard error	Mean	Standard error	
Controls	1.42	0.260	0.497	0.090	0.904
10-Se^IV^	20.0	1.86	7.00	0.650	12.7
10-Se^VI^	49.0	6.58	17.2	2.30	31.2
20-Se^IV^	46.0	7.54	16.1	2.64	29.3
20-Se^VI^	135	14.2	47.4	4.97	86.2

Treatments below indicate the Se application rate (10 and 20 g ha^−1^) and the Se species (selenite (Se^IV^) and selenate (Se^VI^))

The results of this study suggest that wheat biofortification with just 20 g ha^−1^ of Se^VI^ would be a successful strategy for boosting daily per-capita Se intake into the range required to avoid Se deficiency. Chilimba et al. (2012a) estimated that the addition of 5 g ha^−1^ of Se to Malawi soils represented a raw material cost of just 1.6–3.5 US cents ha^−1^ year^−1^.

### Residual Se in the crop at H2 and H3

No ^77^Se_Fert_ could be detected in the first rotation crop (maize) at H2 or the second wheat crop at H3. Thus, within analytical error, all the Se measured in all parts of the maize (grain, husk, leaf, and stem) and wheat (straw, chaff, and grain) was soil-derived. This is consistent with the observation that the major proportion of ^77^Se_Fert_ remaining in the soil had become organically bound (TMAH-extractable) and was unavailable for plant uptake. The TMAH extraction follows extraction with phosphate which should have dissolved ‘adsorbed’ forms of Se. Whatever ^77^Se_Fert_ was still available in the soluble and adsorbed fractions was at too low a concentration to measurably contribute to plant uptake. The negligible recovery of residual Se in the following crops is consistent with other investigations. Mathers et al. (2017) observed a negligible amount of residual Se in follow-up crops in his experiment on ^77^Se application to winter wheat at sites in the UK with 10 g ha^−1^ Se^VI^ applications. Stroud et al. (2010b) reported that no significant difference was found in Se concentration of wheat grain from control plots compared to those that were previously treated with 10 or 20 g ha^−1^ of Se as Na_2_SeO_4_. Similarly, Gupta et al. (1993) also reported no effect of residual Se on barley grain Se content in Canada with applications at three different rates (10, 20, and 40 g ha^−1^) of Se^IV^ and Se^VI^, respectively. Chilimba et al. (2012b) reported a very small recovery (0.78% and 2%) of isotopically labelled residual ^74^Se in maize grain at two different field sites in Malawi, treated with 10 g ha^−1 74^Se^VI^ the previous year. In addition, Wang et al. (2017) also reported a 77.9% and 91.2% reduction in Se concentration in wheat and maize grain from plots, which were treated with 30 and 60 g ha^−1^ of Se in previous cropping season in China. Almost all previous studies suggest that, regardless of soil type, virtually all applied Se retained by topsoil is rendered unavailable for uptake by the time of a second crop.

## Conclusions

Both species of ^77^Se_Fert_ (Se^IV^ and Se^VI^) at application rates of 10 and 20 g ha^−1^ increased Se concentration in a wheat crop. However, Se^VI^ was more efficient in increasing wheat grain Se at H1 and produced 33–40% greater recovery compared to Se^IV^. A single application of 20 g ha^−1^ Se^VI^ increased Se concentration in wheat grain to 135 µg kg^−1^ compared to an extremely low Se concentration of 1.42 µg kg^−1^ from control plots. Considering an average per-capita wheat consumption of 350 g day^−1^ in Pakistan, 20 g ha^−1^ Se^VI^ would provide c. 47 µg person^−1^ day^−1^ of dietary Se, which would be enough to avoid Se deficiency in the Gilgit-Baltistan population. There was no ^77^Se_Fert_ detected in subsequent harvests: H2 (maize) and H3 (wheat) crops suggesting continued annual applications of Se_Fert_ would be required to sustain viable biofortification.

On average, 71.1 ± 16.9% of ^77^Se_Fert_ was lost from the soil at the time of final soil sampling (H3-July 2019). There was no evidence of ^77^Se_Fert_ assimilation into a recalcitrant mineral phase within the timescale of the trial despite clear evidence of native recalcitrant Se. However, the ^77^Se_Fert_ which remained in soil after every harvest was largely (≥ 90%) present in an organically bound form (TMAH extractable) which rendered it completely unavailable for plant uptake so that ^77^Se_Fert_ was not detected in H2 and H3 crops. Therefore, it is clear that Se application to crops in every growing season would be required to obtain an increased concentration of Se in crops. Further research is required to more fully assess (1) the effect of Se lost from the soil on water quality immediately following application and (first) irrigation and (2) any longer-term residual effects from repeated applications as the capacity of these soils to continue fixing Se into organic forms is unknown.

## Supplementary Information

Below is the link to the electronic supplementary material.

Supplementary file1 (DOCX 799 kb)

## Data Availability

The authors confirm that the summary of data supporting the findings of this study are available within the article. However, detailed data of this study are available from corresponding author upon request.
