# Supplementary material for: Fate of selenium in biofortification of wheat on calcareous soil: an isotopic study
Source: Environ Geochem Health. 2021 Feb 25;43(9):3643–57. doi: 10.1007/s10653-021-00841-1 (PMC8405469; doi:10.1007/s10653-021-00841-1)
Supplement: Supplementary file 1 — Supplementary file1 (DOCX 799 kb) [file 10653_2021_841_MOESM1_ESM.docx]

# Supplementary material

**Fate of selenium in biofortification of wheat on calcareous soil: an isotopic study**

Saeed Ahmad^1^, Elizabeth H. Bailey^1*^, Muhammad Arshad^2^, Sher Ahmed^2^, Michael J. Watts^3^, Scott D. Young^1^

^1^Division of Agricultural and Environmental Sciences, School of Biosciences, University of Nottingham, Sutton Bonington Campus, Loughborough, Leicestershire LE12 5RD, United Kingdom

^2^Pakistan Agricultural Research Council – Mountain Agricultural Research Centre, Gilgit-Baltistan, Pakistan

^3^Centre for Environmental Geochemistry, Inorganic Geochemistry, British Geological Survey, Nottingham NG12 5GG, United Kingdom

*Corresponding author: [liz.bailey@nottingham.ac.uk](mailto:liz.bailey@nottingham.ac.uk)


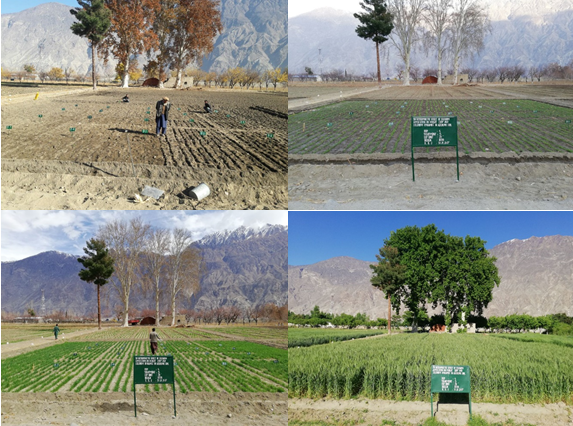


Fig. A1 The field trial site at the Mountain Agriculture Research Centre (MARC) Gilgit station.

| a) | b) |
| --- | --- |
| c) | d) |

Fig. A2 Fractionation of Se_Nat_ in soil representing proportional (%) distribution of Se_Nat_ fractions in soil, a) pre experiment, b) wheat harvest –H1, c) maize harvest – H2 and d) repeat wheat harvest –H3. Error bars represent standard error of means (n=4).

| a) | b) | c) |
| --- | --- | --- |

Fig. A3 Fractionation of ^77^Se_Fert_ in soil representing proportional (%) distribution of ^77^Se_Fert_ fractions in soil after (a) wheat harvest –H1, (b) maize harvest – H2 and (c) repeat wheat harvest –H3 and. Error bars represent standard error of means (n=4)

Table B1. Concentration of selenite (Se^IV^) and selenate (Se^VI^) in soluble and adsorbed fractions of fertilizer derived Se (^77^Se_Fert_) in soil after Harvest 1 (H1)

| *Treatment types and level of Se application | Soluble fraction | |  | Adsorbed fraction | |
| --- | --- | --- | --- | --- | --- |
|  | Se^IV^ | Se^VI^ |  | Se^IV^ | Se^VI^ |
|  | (µg kg^-1^) | |  | (µg kg^-1^) | |
| 10-Se^IV^ | 0.001 ± 0.001 | <LOD |  | 0.036 ± 0.011 | <LOD |
| 10-Se^VI^ | 0.002 ± 0.001 | <LOD |  | 0.022 ± 0.005 | <LOD |
| 20-Se^IV^ | 0.234 ± 0.059 | <LOD |  | 0.032 ± 0.010 | 0.025 ± 0.051 |
| 20-Se^VI^ | 0.123 ± 0.021 | 0.085 ± 0.170 |  | 0.025 ± 0.008 | <LOD |

* The numbers (10 and 20) before the treatment types represent the level of application (g ha^-1^).

Table B2. Change in fractionation of Se_Nat_ in soils at: H0 (pre harvest), H1 (first wheat harvest), H2 (maize harvest) and H3 (second wheat harvest)

| *Treatments and level of application (g ha^-1^) | Soluble (g ha^-1^) | | | | Adsorbed (g ha^-1^) | | | | Organically bound (g ha^-1^) | | | | Recalcitrant (g ha^-1^) | | | |
| --- | --- | --- | --- | --- | --- | --- | --- | --- | --- | --- | --- | --- | --- | --- | --- | --- |
|  | H0 | H1 | H2 | H3 | H0 | H1 | H2 | H3 | H0 | H1 | H2 | H3 | H0 | H1 | H2 | H3 |
| Control | 1.7 | 3.30 | 1.87 | 0.160 | 1.15 | 3.15 | 1.29 | 2.16 | 88.1 | 91.7 | 75.0 | 59.0 | 55.0 | 48.0 | 68.0 | 85.0 |
| 10-Se^IV^ | 1.69 | 3.21 | 1.89 | 0.252 | 1.20 | 2.97 | 1.35 | 2.16 | 90.5 | 89.2 | 77.3 | 60.9 | 51.0 | 49.0 | 64.0 | 81.0 |
| 10-Se^VI^ | 1.58 | 3.23 | 1.85 | 0.268 | 1.12 | 2.13 | 1.30 | 2.06 | 87.8 | 83.9 | 72.6 | 57.5 | 55.0 | 56.0 | 69.0 | 85.0 |
| 20-Se^IV^ | 1.52 | 3.16 | 1.87 | 0.386 | 1.10 | 2.29 | 1.30 | 2.20 | 86.6 | 84.5 | 72.8 | 63.3 | 48.0 | 47.0 | 61.0 | 71.0 |
| 20-Se^VI^ | 1.55 | 3.43 | 1.87 | 0.418 | 1.04 | 2.50 | 1.33 | 2.08 | 86.4 | 92.5 | 75.0 | 61.1 | 59.0 | 49.0 | 70.0 | 84.0 |

*The numbers (10 and 20) before the treatment types represent the level of application (g ha^-1^).

Table B3. Change in fractionation of ^77^Se_Fert_ in soils at: H1 (first wheat harvest), H2 (maize harvest) and H3 (second wheat harvest)

| *Treatments and level of application  (g ha^-1^) | Total ^77^Se_Fert_ remaining in soil  (g ha^-1^) | | | Soluble  (g ha^-1^) | | | Adsorbed  (g ha^-1^) | | | Organically bound  (g ha^-1^) | | | Recalcitrant  (g ha^-1^) | | |
| --- | --- | --- | --- | --- | --- | --- | --- | --- | --- | --- | --- | --- | --- | --- | --- |
|  | H1 | H2 | H3 | H1 | H2 | H3 | H1 | H2 | H3 | H1 | H2 | H3 | H1 | H2 | H3 |
| 10-Se^IV^ | 6.37 | 4.15 | 1.81 | 0.545 | 0.17 | 0.146 | 0.34 | 0.112 | 0.096 | 7.07 | 4.15 | 2.07 | <LOD | <LOD | <LOD |
| 10-Se^VI^ | 5.82 | 4.29 | 3.14 | 0.498 | 0.215 | 0.251 | 0.365 | 0.135 | 0.176 | 5.21 | 4.26 | 3.04 | <LOD | <LOD | <LOD |
| 20-Se^IV^ | 13.9 | 9.75 | 8.02 | 1.02 | 0.41 | 0.503 | 0.849 | 0.292 | 0.37 | 13.7 | 10.1 | 7.07 | <LOD | <LOD | <LOD |
| 20-Se^VI^ | 12.4 | 7.61 | 5.17 | 0.978 | 0.392 | 0.577 | 0.501 | 0.265 | 0.372 | 10.3 | 7.87 | 6.85 | <LOD | <LOD | <LOD |

*The numbers (10 and 20) before the treatment types represent the level of application (g ha^-1^).
